# Effect of intrathecal injection of mesenchymal stem cell-neural progenitors on cerebrospinal fluid biomarkers in progressive multiple sclerosis

**DOI:** 10.1093/stcltm/szag083

**Published:** 2026-09-18

**Authors:** Violaine K Harris, Andrea Jiang, Sofia Ricciarini, Nikki Jagid, Maureen McCormick, Cara Kizilbash, Saud A Sadiq

**Affiliations:** Tisch Multiple Sclerosis Research Center of New York, New York, NY 10019, United States; Tisch Multiple Sclerosis Research Center of New York, New York, NY 10019, United States; Tisch Multiple Sclerosis Research Center of New York, New York, NY 10019, United States; Tisch Multiple Sclerosis Research Center of New York, New York, NY 10019, United States; Tisch Multiple Sclerosis Research Center of New York, New York, NY 10019, United States; Tisch Multiple Sclerosis Research Center of New York, New York, NY 10019, United States; Tisch Multiple Sclerosis Research Center of New York, New York, NY 10019, United States

**Keywords:** multiple sclerosis, mesenchymal stem cells, autologous stem cell transplantation, biomarkers, cerebrospinal fluid

## Abstract

Mesenchymal stem cell-neural progenitors (MSC-NP) are a bone marrow mesenchymal stem cell–derived population of cells with trophic and immunomodulatory properties with therapeutic potential in multiple sclerosis (MS). Early phase clinical trials have investigated the safety and efficacy of intrathecal administration of autologous MSC-NPs in people with progressive MS. To better understand the biological response to MSC-NP treatment, we analyzed cerebrospinal fluid (CSF) biomarkers in 2 separate cohorts of trial subjects with secondary progressive or primary progressive MS from both a phase 2 trial (*n* = 50) and an expanded access trial (*n* = 43) who received repeated administrations of autologous MSC-NPs. Candidate biomarkers identified through proteomic screening were validated in both cohorts, revealing a panel of 4 biomarkers (CCL2, C-C motif chemokine ligand-2; MMP9, matrix metalloproteinase-9; SCF, stem cell factor/c-kit ligand; and CHIT1, chitotriosidase-1) that were significantly changed in CSF but not serum following treatment. Other MS biomarkers neurofilament light and glial fibrillary acidic protein were unchanged following treatment but correlated with age, and both age and Expanded Disability Status Scale (EDSS), respectively. The specific biomarker changes observed following MSC-NP injections suggest distinct biological effects following MSC-NP treatment. These biomarkers help define the pharmacodynamic response to MSC-NP treatment as well as guide the design of future clinical studies.

## Graphical abstract

**Graphical Abstract:**
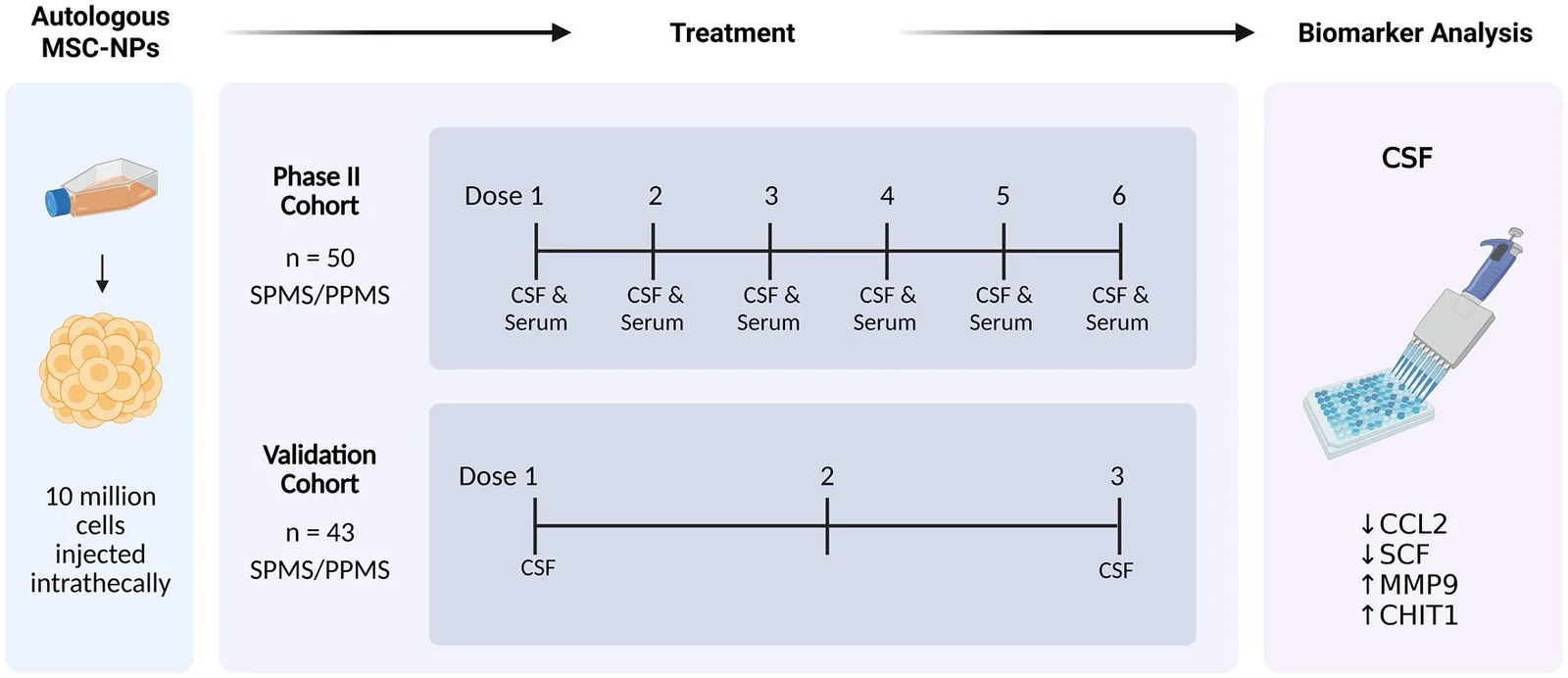

Significance statement Autologous mesenchymal stem cell–derived neural progenitors are currently being investigated as a regenerative approach to treat progressive multiple sclerosis. The aim of the study was to identify biomarkers that correlate with the biological response to stem cell treatment. Analysis from 2 independent cohorts of progressive multiple sclerosis subjects treated with stem cells revealed a panel of 4 novel biomarkers that were consistently altered in the cerebrospinal fluid in response to treatment. The newly identified biomarkers provide insight into the underlying biological mechanisms of this treatment and may serve as surrogate markers in future clinical trials.

## Introduction

Multiple sclerosis (MS) is a chronic immune-mediated disease of the central nervous system leading to neurological deficits caused by neuroinflammation and demyelination. The clinical success in managing disease activity in relapsing remitting MS (RRMS) through access to high-efficacy disease modifying treatments underscores the therapeutic gap in addressing disease progression and the associated accumulation of neurological disability characteristic of secondary or primary progressive MS (SPMS and PPMS, respectively).

Mesenchymal stem cell (MSC)–based therapeutic approaches have been widely studied as a potential treatment for progressive MS based on their immunomodulatory and neuroprotective properties.^1^ In particular, bone marrow MSC-derived neural progenitor-like cells (MSC-NPs), which demonstrate enriched expression of neural and cell signaling molecules and mediate immunomodulatory and trophic effects in preclinical models,^2–5^ have shown promising therapeutic effects in clinical trials.^6–8^ In a recent phase 2 placebo-controlled trial in subjects with progressive MS, multiple intrathecal (IT) injections of autologous MSC-NPs resulted in improved walking outcomes in subjects with MS requiring assistance for ambulation, as well as improved bladder function and reduced grey matter atrophy.^7^

Given the limited sensitivity of standard clinical outcomes in progressive MS and the inherent heterogeneity in both MSC potency and patient biology, there is a critical need for objective biomarkers that can sensitively detect biological activity and reliably correlate with the therapeutic effects of MSC-based treatment. Two previous clinical trials in MS have identified the proinflammatory chemokine CCL2 as a candidate biomarker that is decreased in cerebrospinal fluid (CSF) following IT-MSC-NP treatment.^7^^,^^8^ Additionally, MSC or MSC-NP treatment was associated with CSF changes in MMP9, CXCL13, and NfL, thus identifying additional candidate biomarkers of IT cell therapy.^7^^,^^9^

The aim of the current study is to investigate biomarkers of MSC-NP treatment identified via an unbiased proteomic screening of CSF samples before and after MSC-NP injection.^7^ We report the effects of MSC-NP treatment on a panel of biomarkers and demonstrate validation of biomarker changes in an independent cohort of subjects with progressive MS.

## Materials and methods

### Sample information

Samples were obtained from 2 separate trial cohorts. Both trials were approved by WCG IRB and all subjects gave written informed consent to participate. The phase 2 clinical trial (NCT03355365) cohort (*n* = 50) consisted of subjects with SPMS or PPMS enrolled following inclusion/exclusion criteria including confirmed nonactive progressive disease defined by lack of EDSS change, absence of relapses, and stable MRI in the year prior to enrollment.^7^ Subjects were treated with 6 separate IT-MSC-NP injections every 2 months.^7^ The validation cohort (*n* = 43) consisted of SPMS and PPMS subjects participating in an expanded access program (NCT03822858) who received 3 separate IT-MSC-NP injections every 3 months. Subject demographics are shown in Table 1. All subjects received a dose of up to 20 million autologous MSC-NPs manufactured in a cGMP facility from bone marrow as described previously.^7^

**Table 1.** Patient demographics.

	Phase 2 trial cohort (*n* = 50)	Expanded access cohort (*n* = 43)
**Female, *n* (%)**	36 (72%)	30 (70%)
**SPMS, *n* (%)**	39 (78%)	34 (79%)
**PPMS, *n* (%)**	11 (22%)	9 (21%)
**Age (years), mean (range)**	50 (31-65)	58 (31-76)
**EDSS, median (range)**	5.3 (3.0-6.5)	6.5 (2.0-8.5)

Abbreviations: PPMS, primary progressive multiple sclerosis; EDSS, expanded disability status scale; SPMS, secondary progressive multiple sclerosis.

In the phase 2 cohort, CSF and serum were collected at each IT procedure just prior to cell or saline injection. In the expanded access cohort, CSF was collected at the first and third IT-MSC-NP treatment. Cell-free CSF and serum samples were processed immediately and stored at −80 °C. Pretreatment/baseline was defined by CSF collected prior to the first injection. Post-treatment was defined by CSF collected before the final injection (after 5 previous doses in the phase 2 cohort, and after 2 previous doses in the expanded access cohort).

### Biomarker analysis

Proteomic analysis of CSF was performed using Slow Off-rate Modified Aptamers assay (SOMAScan Assay v4·1, SomaLogic, Inc) as previously reported.^7^ MMP9 was measured in CSF (undiluted) and serum (1:50 dilution) using human MMP9 magnetic Luminex performance assay (R&D Systems). CCL2 and SCF were measured in CSF (undiluted) and serum (1:4 dilution) using Bio-Plex Pro human cytokine panel (Bio-Rad). CHIT1 (chitotriosidase-1) was measured in CSF (1:5 dilution) and serum (1:50 dilution) using human Chitotriosidase ELISA (Circulex, CY-8074). NfL was measured in CSF (1:1 dilution) and serum (1:4 dilution) using NF-light™ CSF ELISA (Tecan, 30112458) and NF-light™ Serum ELISA (Tecan, 30210101), respectively. GFAP was measured in CSF (undiluted) using Milliplex human Neuroscience Magnetic Beads (Millipore Sigma, GFAP-HNS1MAG-95K01). C3a was measured in CSF (undiluted) using Microvue Complement C3a Plus EIA (Quidel). Gal-1 and ICAM-1 were measured in CSF (undiluted) using magnetic Luminex discovery assay (R&D Systems). Luminex assays were analyzed using Bio-Plex Pro 200 system (Bio-Rad) and ELISAs were analyzed using Synergy HTX plate reader (Agilent).

### Statistical analysis

Differentially expressed proteins were identified from proteomic analysis using a multivariate linear mixed effect model with patient age and EDSS modeled as a continuous covariate and other independent variables (treatment, diagnosis, and gender) as discrete. The model was performed using R version 4.0.3. Pairwise contrasts (post-treatment minus pretreatment) were extracted with least squares means function and *P*-values were adjusted for multiple comparisons using the Benjamini–Hochberg false discovery rate (FDR) method. Statistical significance for differential expression was defined as FDR q ≤ 0.05. Candidate exploratory biomarkers were selected for downstream validation based on nominal *P*-values (*P* < .01) and potential biological relevance to MS. Differences between biomarker levels before and after treatment were analyzed using Wilcoxon matched-pairs signed rank test. Longitudinal biomarker measurements were analyzed using a mixed-effects model fitted by restricted maximum likelihood with treatment visit as a fixed effect and subject as a random effect to account for repeated measurements. When the overall effect of treatment visit was significant, each post-treatment visit was compared with baseline using Sidak’s multiple-comparisons correction. Correlations were determined by simple linear regression analysis. Statistical significance was set to a *P* value of <.05. Graphpad Prism 10 was used to calculate significance.

## Results

Candidate biomarkers of IT-MSC-NP treatment were identified through a discovery-phase proteomic analysis of CSF collected from 36 phase 2 trial participants pretreatment (prior to the first injection) and post-MSC-NP treatment (at the time of treatment 6). Three proteins (MMP9, MMP12, and CHIT1) were identified as significantly differentially expressed (FDR q ≤ 0.05). In addition, 144 proteins demonstrating nominal significance (*P* < .01) were considered exploratory.^7^ From this set, 19 candidate biomarkers were selected for validation based on a combination of nominal *P*-values and biological relevance to neuroinflammation and MS (Figure S1). Of the 19 proteins, 9 (CCL22, MMP12, MMP8, TNFSF14, CD79A, IL-34, WISP-1, NSF, and NPY) were not detectable in CSF by standard immunoassay. Galectin-1, ICAM-1, and complement protein C3a were assayed in the phase 2 cohort but there was no significant difference found following treatment (Figure S2).

**Figure 1.**
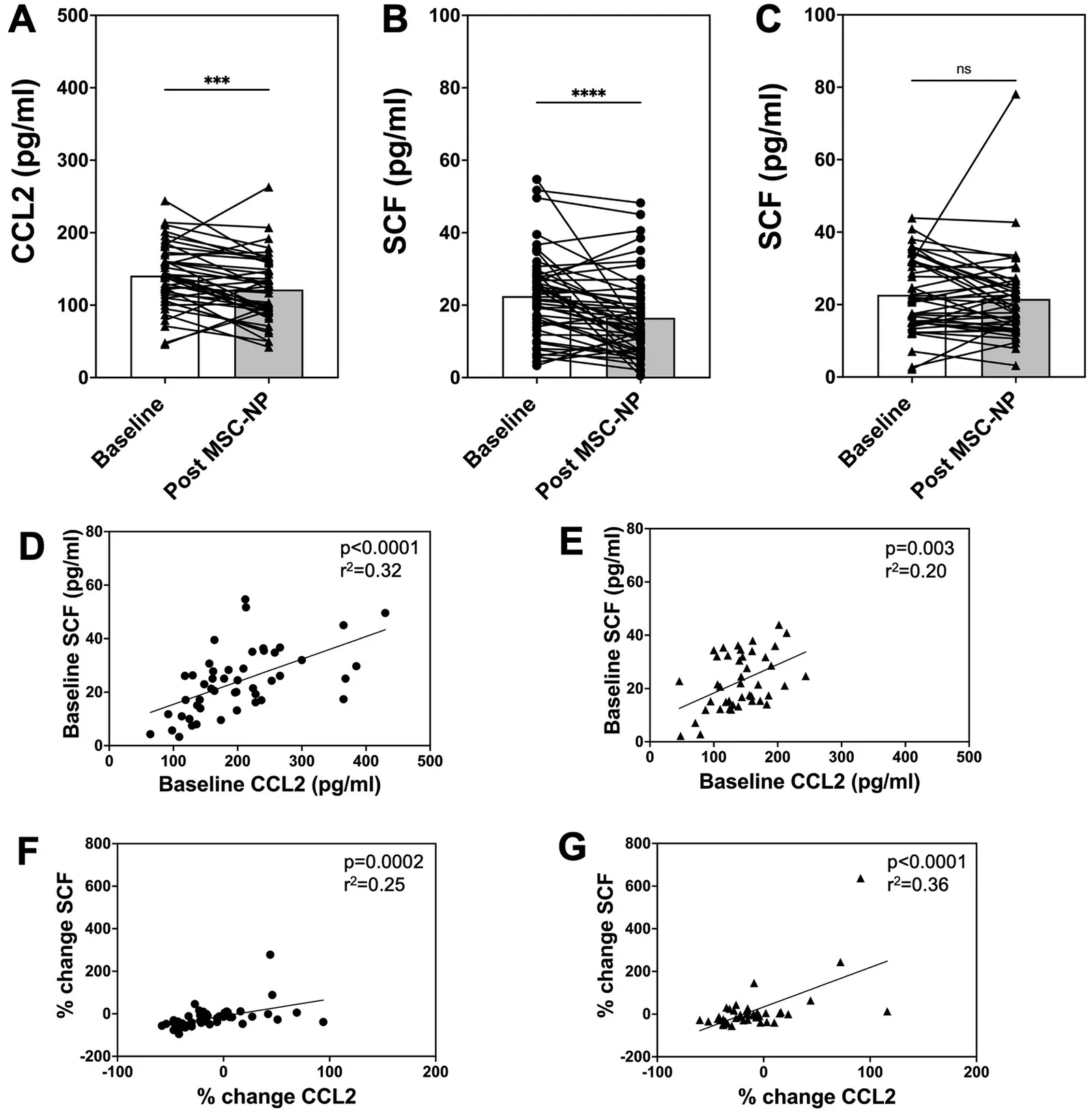
Decreased CCL2 and SCF levels in CSF following MSC-NP treatment. (A) CCL2 levels in CSF pre- and post-MSC-NP treatment in the expanded access cohort. (B, C) SCF levels in CSF pre- and post-MSC-NP treatment in the (B) phase 2 and (C) expanded access cohorts. Pretreatment sample was collected prior to the first injection, and post-treatment sample was collected prior to the final injection (after 5 previous doses in phase 2, and after 2 previous doses in expanded access). (D, E) Baseline values of CCL2 and SCF in CSF significantly correlate with each other in both the (D) phase 2 and (E) expanded access cohorts. (F, G) Percentage change of CCL2 levels in CSF following MSC-NP treatment significantly correlates with change in SCF levels in both the (F) phase 2 and (G) expanded access cohorts. Solid circles and triangles represent individual values in the phase 2 and expanded access cohorts, respectively. Bars represent mean value. ***, *P* < .001; ****, *P* < .0001. “ns,” not significant.

**Figure 2.**
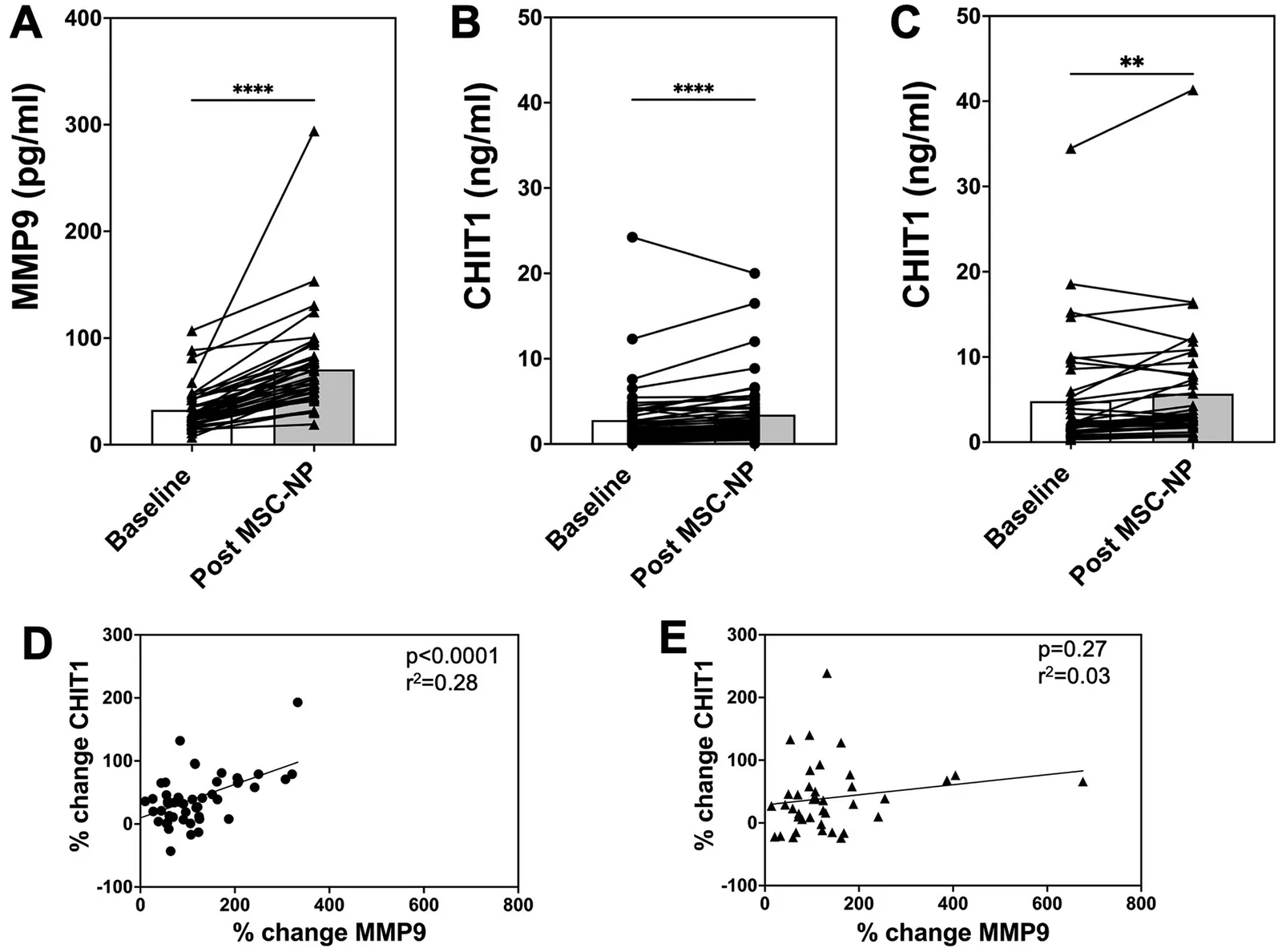
Increased MMP9 and CHIT1 levels in CSF following MSC-NP treatment. (A) MMP9 levels in CSF pre- and post-MSC-NP treatment in the expanded access cohort. (B, C) CHIT1 levels in CSF pre- and post-MSC-NP treatment in the (B) phase 2 and (C) expanded access cohorts. Pretreatment sample was collected prior to the first injection, and post-treatment sample was collected prior to the final injection (after 5 previous doses in phase 2, and after 2 previous doses in expanded access). (D, E) Percentage change of MMP9 levels in CSF following MSC-NP treatment significantly correlates with change in CHIT1 levels in the (D) phase 2 cohort but not the (E) expanded access cohort. Solid circles and triangles represent individual values in the phase 2 and expanded access cohorts, respectively. Bars represent mean value. **, *P* < .01; ****, *P* < .0001.

Previous validation by immunoassay confirmed a significant decrease in CCL2 and increase in MMP9 levels in post-treatment CSF (sampled at treatment 6 following 5 previous treatments) compared to baseline in the phase 2 cohort.^7^ Longitudinal analysis of CCL2 and MMP9 at each treatment demonstrated significant changes after 1 treatment that gradually decreased or increased over time, respectively (Figure S3A and B). These findings were confirmed in the expanded access cohort demonstrating a decrease in CCL2 (Figure 1A) and increase in MMP9 occurring after 2 IT-MSC-NP treatments (Figure 2A).

**Figure 3.**
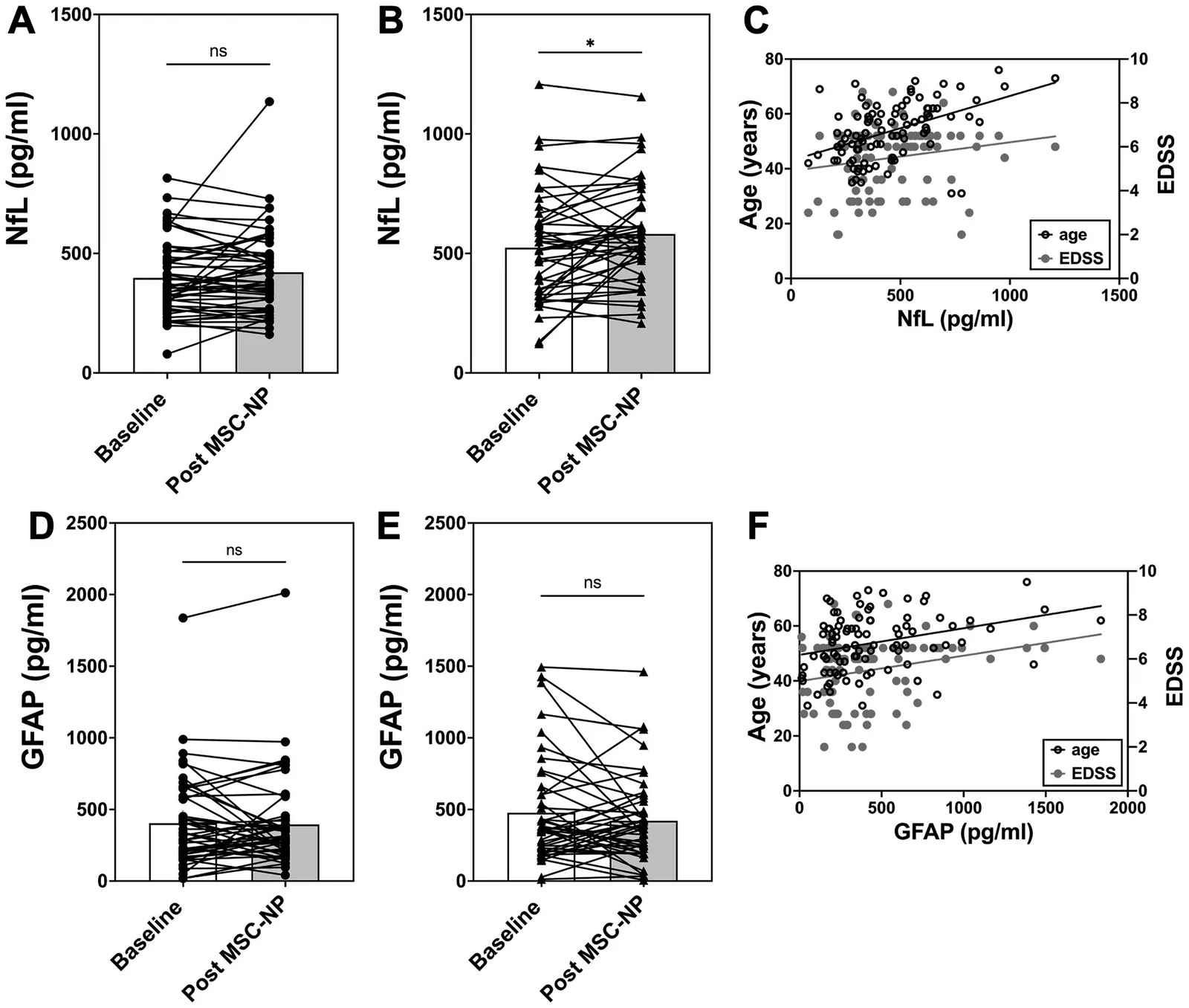
No effect of MSC-NP treatment on NfL or GFAP in CSF. (A, B) NfL levels in CSF pre- and post-MSC-NP treatment were (A) unchanged in the phase 2 cohort and (B) slightly elevated in the expanded access cohort. (C) Baseline CSF levels of NfL correlated with age (*P* < .0001) but not EDSS (combined phase 2 and expanded access cohorts). (D, E) GFAP levels in CSF pre- and post-MSC-NP treatment were unchanged in both the (D) phase 2 and (E) the expanded access cohorts. (F) Baseline CSF levels of GFAP correlated with age (*P* = .002) and EDSS (*P* = .01) (combined phase 2 and expanded access cohorts). Solid circles and triangles represent individual values in the phase 2 and expanded access cohorts, respectively. Bars represent mean value. *, *P* < .05. “ns,” not significant.

SCF (stem cell factor) was identified in the proteomic screen as a candidate biomarker that was decreased in CSF following treatment (Figure S1). Validation of SCF levels in the full phase 2 cohort demonstrated a significant decrease in CSF following 5 IT-MSC-NP treatments compared to baseline (Figure 1B). In the expanded access cohort, the decrease in SCF levels did not reach significance (Figure 1C) possibly due to the slower temporal dynamics of SCF decrease over the treatment course (Figure S3C). Nevertheless, both cohorts demonstrated a correlation between SCF and CCL2 baseline values (Figure 1D and E) and the percent change (Figure 1F and G) suggesting a relationship between the 2 biomarkers.

CHIT1 was identified as a candidate biomarker that was increased in CSF following MSC-NP treatment (Figure S1). Validation of CHIT1 levels in CSF in both cohorts confirmed a significant increase in CHIT1 following treatment (Figure 2B and C), with a gradual increase in CHIT1 with each treatment in the phase 2 cohort (Figure S3D and E). The percent change of CHIT1 and MMP9 was highly correlated in the phase 2 cohort (Figure 2D) suggesting that the degree of increase in both biomarkers after treatment followed the same pattern. In contrast, the percent change of CHIT1 and MMP9 did not correlate in the expanded access cohort (Figure 2E) possibly reflecting the shorter duration of treatment in this cohort (2 treatments) compared to the phase 2 cohort (5 treatments). Baseline levels of MMP9 and CHIT1 did not correlate in either cohort (data not shown).

To confirm that the change in biomarkers was not a procedure-related effect, we analyzed biomarker levels following IT injection of saline in the subgroup of phase 2 subjects (*n* = 26) that received placebo in the year prior to crossover into the treatment arm.^7^ Previous analysis demonstrated that levels of MMP9 and CCL2 remained stable following saline injections.^7^ Similarly, SCF levels remained stable (Figure S4A) and CHIT1 levels decreased (Figure S4B) following saline injections. These results suggest that the biomarker changes following MSC-NP injections reflect a biologic response to treatment rather than a consequence of repeated IT injection procedures.

In addition to the candidate biomarkers discovered by proteomic screening, we tested NfL and GFAP which are widely studied biomarkers of MS disease activity and progression, respectively.^10^ NfL levels were unchanged in the phase 2 cohort (Figure 3A) and were slightly elevated in the expanded access cohort (Figure 3B). Of note, only 1 patient in each cohort showed elevated levels of NfL (>1000 pg/ml) confirming the lack of disease activity in this non-active progressive MS patient population.^11^ Testing of GFAP levels in CSF also demonstrated a lack of change following treatment in either cohort (Figure 3D and E). Despite the lack of correlation between GFAP or NfL levels and MSC-NP treatment, baseline levels of NfL levels correlated with age (Figure 3C), and GFAP levels correlated with age and EDSS and (Figure 3F), as expected.^10^ None of the other biomarkers reported in this study showed any correlation with age or EDSS (data not shown).

Biomarker levels were also tested in serum samples collected in the phase 2 cohort only. Serum levels of CCL2, MMP9, SCF, CHIT1, and NfL did not significantly change post-treatment compared to baseline (GFAP was undetectable in serum by standard ELISA) (Figure S5A through E). Furthermore, there was no correlation between CSF and serum levels at baseline or post-treatment, or percent change in levels from baseline to post-treatment in any of the biomarkers (CCL2, MMP9, SCF, CHIT1, or NfL) (Figure S5F-O). Although these findings were limited to the phase 2 cohort and may have been impacted by differences in assay sensitivity and matrix-dependent detectability, the lack of effect on serum levels suggests that biomarker changes were specific to CSF following IT treatment.

## Discussion

The current study identifies a novel panel of 4 biomarkers, CCL2, MMP9, SCF, and CHIT1, that are significantly altered in CSF following IT injection of autologous MSC-NPs in patients with progressive MS. Remarkably, biomarker changes were consistent in 2 independent patient cohorts, 1 of which received a shorter duration of treatment with CSF sampling after 2 treatments rather than 5. The biomarker changes in response to MSC-NP but not saline injections point to specific biological effects induced by MSC-NP treatment, offering insight into its underlying therapeutic mechanisms and informing strategies for treatment refinement.

CCL2 is a proinflammatory chemokine with increased expression in microglia and astrocytes in MS and encephalomyelitis (EAE) lesions where it is associated with recruitment of peripheral monocytes and the exacerbation of demyelination.^12^ The observation that CCL2 is decreased in CSF following IT administration of MSC-NPs or MSCs is consistent across multiple studies, suggesting a shared biological effect of IT cell therapy in both MS and ALS.^7^^,^^8^^,^^13^^,^^14^ A previous study demonstrated that MSC-NPs dampen microglial activation in vitro,^15^ thus we hypothesize that IT-MSC-NP administration may be targeting activated microglia that play a known role in MS progression. The role of SCF, which closely correlated with CCL2, is less known in MS. A previous study that performed cytokine profiling in newly diagnosed MS patients showed that SCF levels in CSF were found to be elevated along with other proinflammatory cytokines.^16^ SCF and its receptor c-kit are expressed in neurons, astrocytes, and microglia in the CNS, and both are upregulated after brain injury.^17^ Although previous studies suggest SCF may modulate microglia activity,^17^^,^^18^ any mechanistic correlation between SCF and CCL2 remains unknown. Interestingly, inhibition of the SCF receptor c-kit by masitinib in PPMS or non-active SPMS patients slowed disease progression.^19^ Further investigation will be required to determine whether SCF plays any role in the neurodegenerative process in progressive MS.

MMP9 and CHIT1 have both been previously identified as CSF biomarkers in early stages of MS, reflecting blood brain barrier breakdown and microglial activation, respectively.^20–22^ Given that MMP9 and CHIT1 have a well-documented correlation with neuroinflammation in RRMS, the treatment-related increase in progressive MS was somewhat unexpected. Previous preclinical studies have demonstrated that MMP9-mediated modulation of the extracellular matrix promotes synaptic remodeling, neurogenesis, and oligodendrocyte maturation during remyelination, suggesting potential mechanisms by which MMP9 may participate in the regenerative process.^23^^,^^24^ Similarly, 1 study demonstrated that CHIT1 promotes oligodendrocyte differentiation of neural stem cells in vitro, and the mouse paralogue Chi3l3 affects disease severity in EAE through a pro-oligodendrogenic mechanism.^25^ Further investigation will be required to understand how the increase in MMP9 and CHIT1 might reflect a biological effect of IT-MSC-NP injection, the cellular source of MMP9 and CHIT1 expression, and whether the 2 biomarkers are mechanistically connected. Elevated CSF MMP9 was also reported following transplantation of neural stem cells in patients with MS, indicating an association with biological responses observed after IT cell therapy.^26^

While NfL and GFAP have been widely studied as MS biomarkers of axonal damage due to disease activity and astrogliosis during disease progression, respectively,^10^ no changes in these biomarkers were observed in response to MSC-NP injection. The lack of change in NfL was consistent across multiple trial cohorts,^8^ and likely reflects the inclusion of non-active SPMS and PPMS subjects with already low levels of CSF NfL. The stable levels of CSF GFAP levels over the course of treatment in both cohorts may signify a lack of impact on astrogliosis. One limitation on the interpretation of these results is that the current study did not use the Simoa-based methods for NfL or GFAP detection which may explain any discrepancies with other studies.^9^^,^^27^

Repeated IT injections have been shown to be a safe and well-tolerated route of administration for the CNS-targeting of therapeutics in people with MS.^6^^,^^7^^,^^28^^,^^29^ In contrast to a recent study,^30^ we found no clinical or laboratory evidence of spinal nerve root abnormalities, back pain, arachnoiditis, or increased cellularity in any of the subjects from either the phase 1, phase 2 or expanded access cohorts, reflecting over 800 separate IT procedures, some of which consisted of saline injections.^6^^,^^7^^,^^28^ In addition, biomarker levels were stable in CSF from placebo-treated trial subjects, suggesting that biomarker changes reflect a specific biologic response to MSC-NP treatment rather than a structural reaction to the IT procedure.^7^ One caveat is the limited number of subjects who received placebo prior to MSC-NPs. Subjects receiving placebo after the crossover were not analyzed due to possible carryover effects of the MSC-NP treatment, and the expanded access cohort did not have a placebo control.

The biomarker panel described in this study was limited to analytes detectable by immunoassay. Discrepancy in analyte detection between the SOMAscan platform and immunoassay may be due to increased detection of low abundance proteins by SOMAmers. However, additional interpretations of non-detectability may be related to platform-specific measurement issues including differences in epitope recognition, detection of protein isoforms, post-translational modifications, protein complexes, and matrix effects that can contribute to discordant results across platforms. Consequently, failure to validate a SOMAscan finding by immunoassay does not necessarily exclude a true biological signal.

## Conclusion

Regenerative therapy using autologous MSC-NPs is an emerging treatment for progressive MS. Clinical trials investigating the efficacy of MSC-based treatments have shown inconsistent efficacy likely due to patient heterogeneity, poorly defined therapeutic mechanisms, and differences in potency of donor cells.^1^ Herein, we identify a panel of 4 analytes that are easily measured in CSF samples and are reproducibly altered in response to IT-MSC-NP treatment suggestive of a biological response to treatment. Although the current study is limited by a relatively low number of subjects and a lack of clear correlation to clinically meaningful outcomes, the identification of novel biomarkers specific to IT-MSC-NP therapy provides important insight into the underlying biological response and offers a potential framework for refining and optimizing this therapeutic approach.

## Supplementary Material

szag083_Supplementary_Data

## Data Availability

The datasets used and/or analyzed during the current study are available from the corresponding author on reasonable request.
