# Supplementary material for: Effect of intrathecal injection of mesenchymal stem cell-neural progenitors on cerebrospinal fluid biomarkers in progressive multiple sclerosis
Source: Stem Cells Transl Med. 2026 Sep 18;15(10):szag083. doi: 10.1093/stcltm/szag083 (PMC13585449; doi:10.1093/stcltm/szag083)
Supplement: Supplementary file 1 [file szag083_supplementary_data.pdf]

SUPPLEMENTARY INFORMATION

Effect of Intrathecal Injection of Mesenchymal Stem Cell-Neural Progenitors on Cerebrospinal Fluid Biomarkers in Progressive Multiple Sclerosis

Running Title: CSF biomarkers of MSC-NP treatment in MS

Authors: Violaine K. Harris,[1] Andrea Jiang,[1] Sofia Ricciarini,[1] Nikki Jagid,[1] Maureen McCormick,[1] Cara Kizilbash,[1] and Saud A. Sadiq[1]

[1]Tisch Multiple Sclerosis Research Center of New York, New York, NY, USA

Corresponding Author:
Violaine Harris, PhD
Tisch Multiple Sclerosis Research Center of New York
521 West 57th Street, 4th floor
New York, NY 10019 USA
Ph: (646) 557-3880
Email: vharris@tischms.org

Supplemental Figure 1

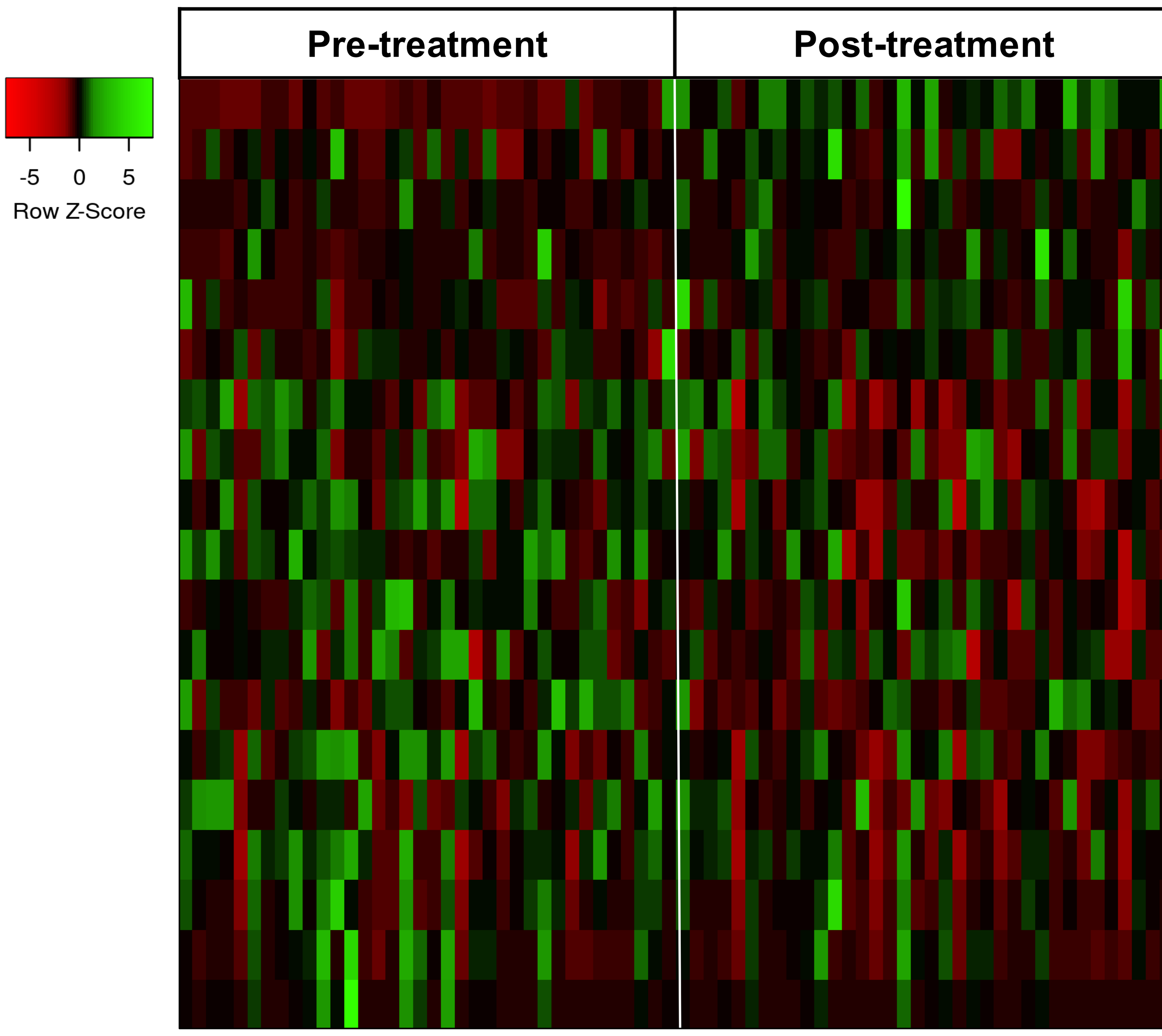

Name	FC (Log2)	p value	FDR q value
MMP9	1.25	7.63×10^{-17}	5.56×10^{-13}
CHIT1	0.31	5.64×10^{-7}	1.37×10^{-3}
CCL22	0.25	6.83×10^{-3}	0.485
MMP12	0.18	3.59×10^{-9}	1.31×10^{-5}
MMP8	0.08	1.18×10^{-3}	0.269
TNFSF14	0.03	5.81×10^{-3}	0.485
Galectin-1	-0.07	6.91×10^{-3}	0.485
ICAM1	-0.07	9.44×10^{-3}	0.485
C3a desArg	-0.07	5.09×10^{-5}	0.0618
CD79A	-0.07	4.78×10^{-5}	0.0618
CCL2	-0.08	8.99×10^{-3}	0.485
IL34	-0.09	7.15×10^{-6}	0.0130
WISP-1	-0.10	2.71×10^{-4}	0.198
C3	-0.10	1.11×10^{-3}	0.269
NSF	-0.11	4.81×10^{-3}	0.438
NPY	-0.13	9.01×10^{-3}	0.485
SCF	-0.13	5.10×10^{-3}	0.442
C3d	-0.31	1.80×10^{-3}	0.328
C3a	-0.61	1.70×10^{-3}	0.318

Supplemental Figure 1. Heatmap of candidate biomarkers following proteomic analysis of CSF. CSF was collected from 36 Phase 2 trial participants pre- and post-MSC-NP treatment. CSF supernatants (n=72) were analyzed using SOMAScan Assay. Heatmap shows relative fluorescent unit values for 19 selected differentially expressed proteins with p value <0.01, listed in order of fold change.

Supplemental Figure 2

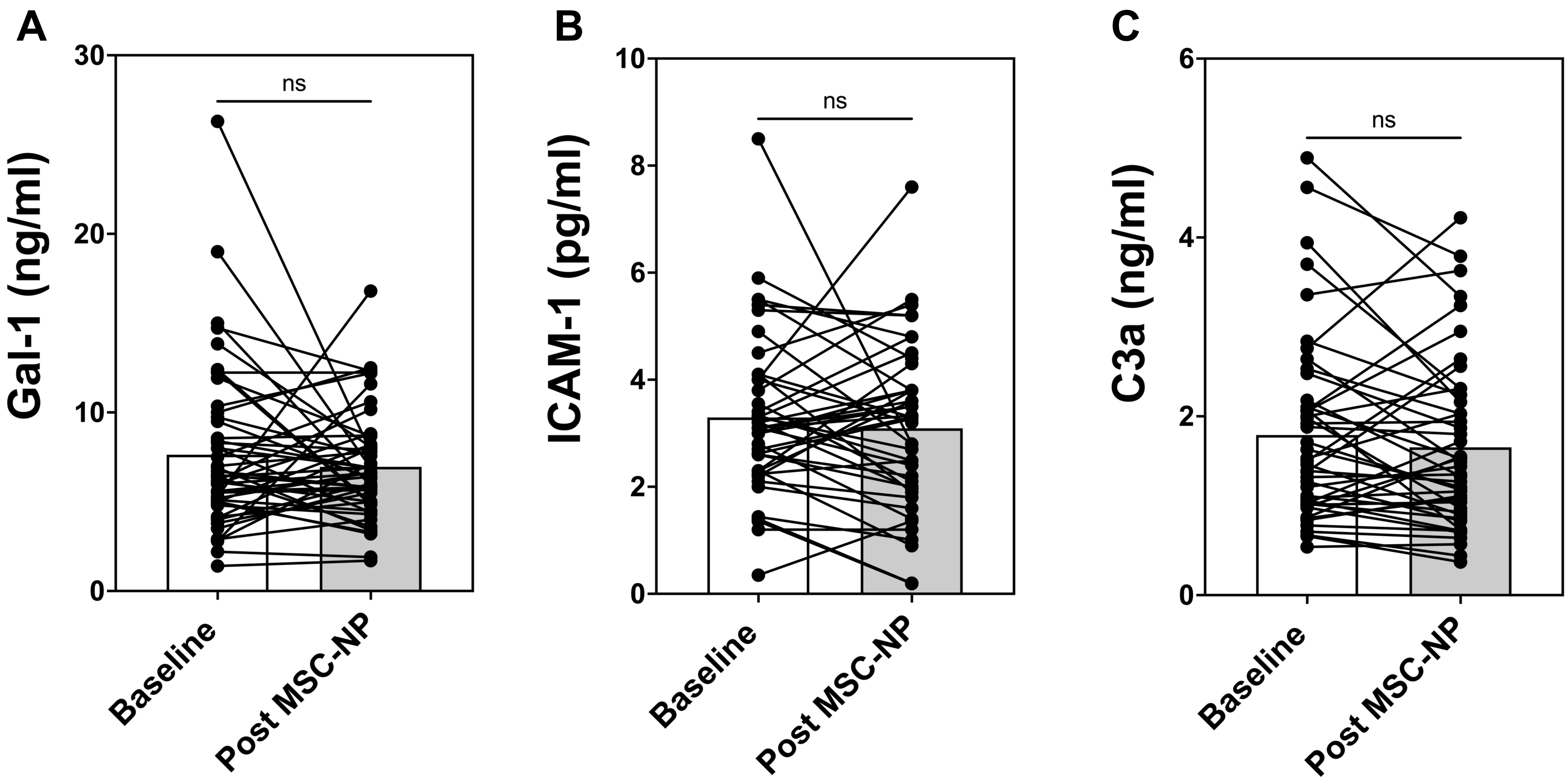

Supplemental Figure 2. No change in Gal-1, ICAM-1, or C3a levels in CSF following MSC-NP treatment. (A) Gal-1, **(B)** ICAM-1, and **(C)** C3a levels in CSF pre- and post-MSC-NP treatment in the phase 2 cohort. Bars represent mean value. "ns", not significant.

Supplemental Figure 3

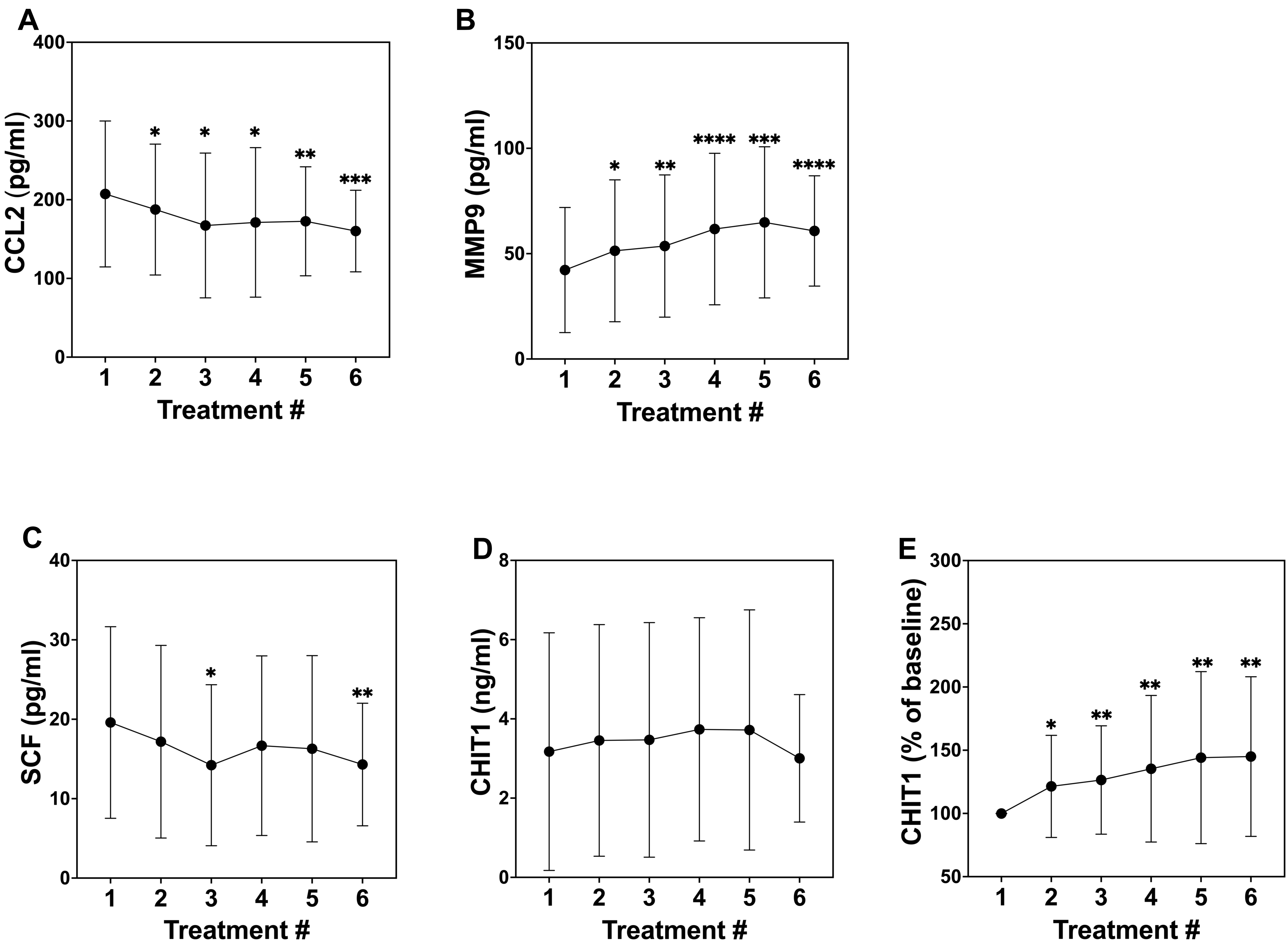

Supplemental Figure 3. Temporal dynamics of biomarker changes in the Phase 2 cohort. **(A)** CCL2, **(B)** MMP9, **(C)** SCF and **(D, E)** CHIT1 were measured longitudinally in samples collected prior to each of 6 IT MSC-NP treatments in the phase 2 cohort. Treatment #1 corresponds to the baseline sample. Data points represent mean and standard deviation of CSF biomarker concentration as indicated in A-D. Due to the baseline inter-individual variability of CHIT1 **(D)**, within-subject treatment responses of CHIT1 concentrations were normalized to each participant's baseline value **(E)**. Following significance of mixed-effects longitudinal modeling (A, B, C and E), post-treatment samples were compared to baseline in post-hoc comparison. *, $p<0.05$; **, $p<0.01$; ***, $p<0.001$; ****, $p<0.0001$.

Supplemental Figure 4

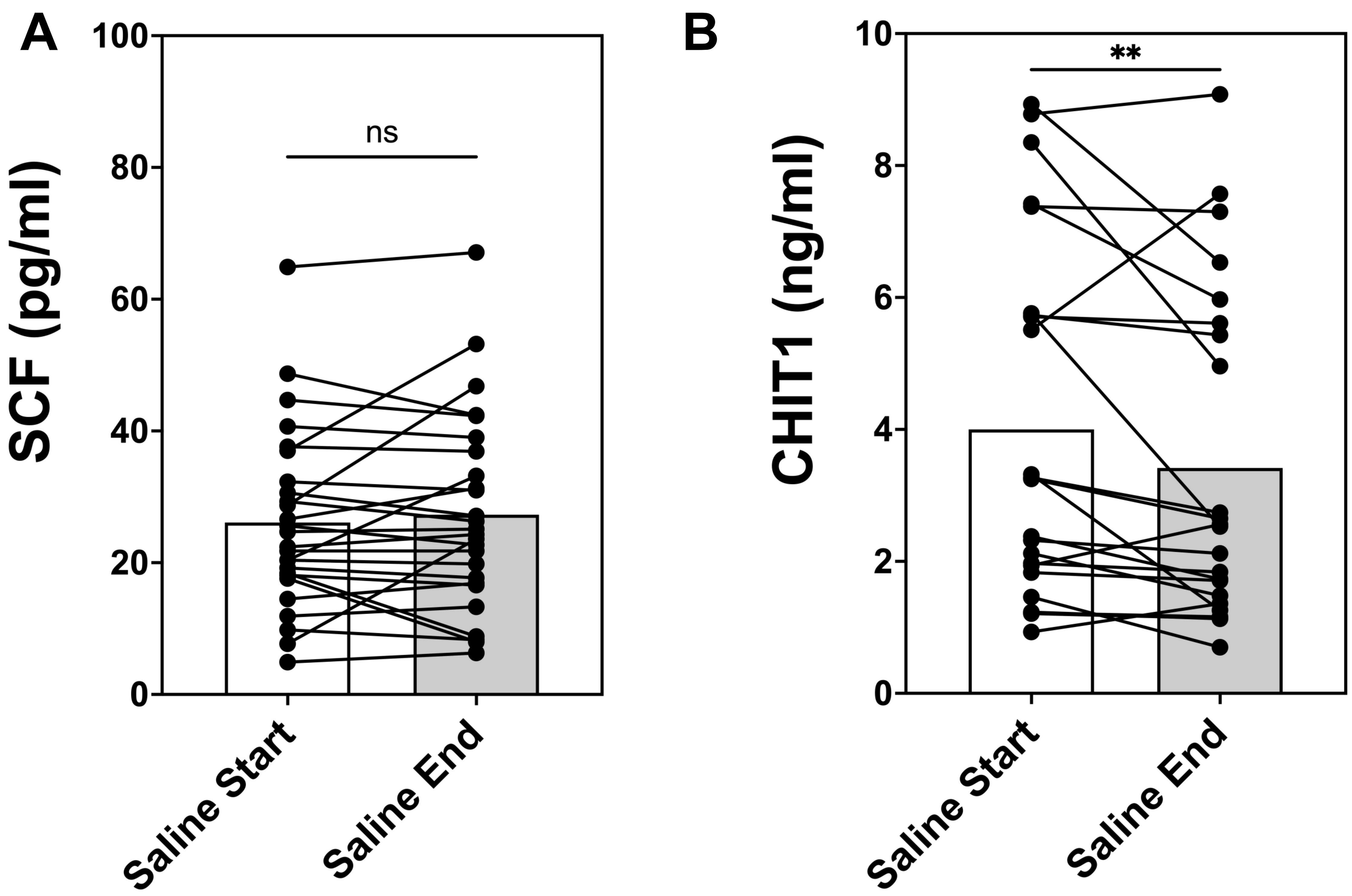

Supplemental Figure 4. SCF and CHIT1 levels in CSF following saline placebo treatment. (A) SCF levels were stable and **(B)** CHIT1 levels decreased rather than increased in CSF following saline treatment in the phase 2 cohort subgroup (n=26) receiving placebo in the year prior to MSC-NP treatments. Bars represent mean value. "ns", not significant, **, p<0.01.

Supplemental Figure 5

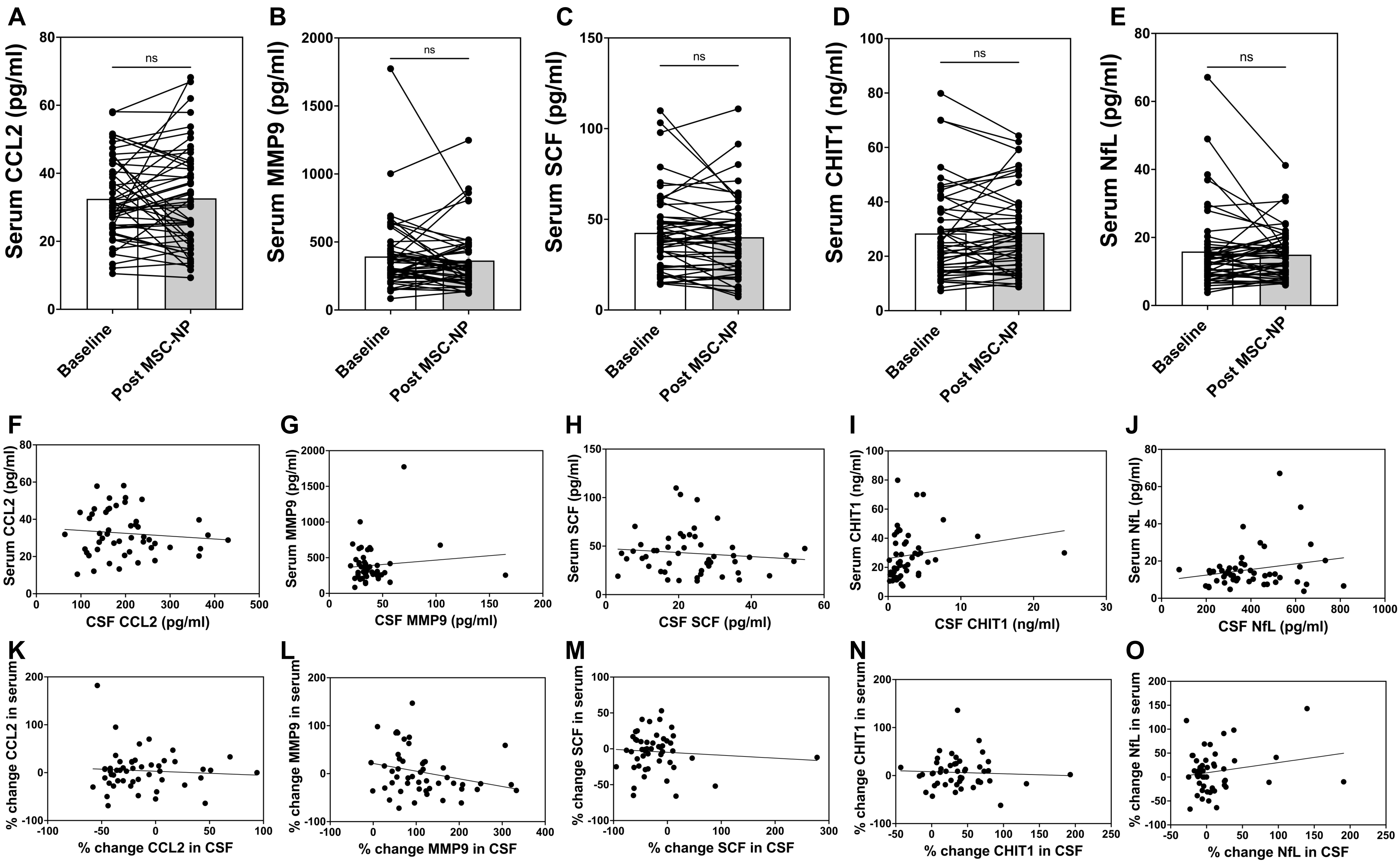

Supplemental Figure 5. No change in serum levels of biomarkers following MSC-NP treatment. (A) CCL2, **(B)** MMP9, **(C)** SCF, **(D)** CHIT1, and **(E)** NfL levels in serum pre- and post-MSC-NP treatment in the phase 2 cohort were unchanged following treatment. **(F-J)** Baseline levels of all biomarkers in CSF did not correlate with levels in serum. All p values were >0.05. **(K-O)** Percentage change of all biomarkers in CSF pre- vs. post-treatment did not correlate with percentage change in serum. All p values were >0.05.
